# Combined quality and dose-volume histograms for assessing the predictive value of ^99m^Tc-MAA SPECT/CT simulation for personalizing radioembolization treatment in liver metastatic colorectal cancer

**DOI:** 10.1186/s40658-020-00345-4

**Published:** 2020-12-14

**Authors:** Hugo Levillain, Manuela Burghelea, Ivan Duran Derijckere, Thomas Guiot, Akos Gulyban, Bruno Vanderlinden, Michael Vouche, Patrick Flamen, Nick Reynaert

**Affiliations:** 1 Medical Physics Department, Jules Bordet Institute, Université Libre de Bruxelles, 1 Rue Héger-Bordet, B-1000 Brussels, Belgium; 2 Nuclear Medicine Department, Jules Bordet Institute, Université Libre de Bruxelles, 1 Rue Héger-Bordet, 1000 Brussels, Belgium; 3 Department of Radiology, Jules Bordet Institute, Université Libre de Bruxelles, 1 Rue Héger-Bordet, 1000 Brussels, Belgium

**Keywords:** Radioembolization, SIRT, mCRC, Dosimetry, MAA, DVH, QVH

## Abstract

**Background:**

The relationship between the mean absorbed dose delivered to the tumour and the outcome in liver metastases from colorectal cancer patients treated with radioembolization has already been presented in several studies. The optimization of the personalized therapeutic activity to be administered is still an open challenge. In this context, how well the ^99m^Tc-MAA SPECT/CT predicts the absorbed dose delivered by radioembolization is essential. This work aimed to analyse the differences between predictive ^99m^Tc-MAA-SPECT/CT and post-treatment ^90^Y-microsphere PET/CT dosimetry at different levels. Dose heterogeneity was compared voxel-to-voxel using the quality-volume histograms, subsequently used to demonstrate how it could be used to identify potential clinical parameters that are responsible for quantitative discrepancies between predictive and post-treatment dosimetry.

**Results:**

We analysed 130 lesions delineated in twenty-six patients. Dose-volume histograms were computed from predictive and post-treatment dosimetry for all volumes: individual lesion, whole tumoural liver (TL) and non-tumoural liver (NTL). For all dose-volume histograms, the following indices were extracted: *D*_90_, *D*_70_, *D*_50_, *D*_mean_ and *D*_20_. The results showed mostly no statistical differences between predictive and post-treatment dosimetries across all volumes and for all indices. Notably, the analysis showed no difference in terms of *D*_mean_, confirming the results from previous studies. Quality factors representing the spread of the quality-volume histogram (QVH) curve around 0 (ideal QF = 0) were determined for lesions, TL and NTL. QVHs were classified into good (QF < 0.18), acceptable (0.18 ≤ QF < 0.3) and poor (QF ≥ 0.3) correspondence. For lesions and TL, dose- and quality-volume histograms are mostly concordant: 69% of lesions had a QF within good/acceptable categories (40% good) and 65% of TL had a QF within good/acceptable categories (23% good). For NTL, the results showed mixed results with 48% QF within the poor concordance category. Finally, it was demonstrated how QVH analysis could be used to define the parameters that predict the significant differences between predictive and post-treatment dose distributions.

**Conclusion:**

It was shown that the use of the QVH is feasible in assessing the predictive value of ^99m^Tc-MAA SPECT/CT dosimetry and in estimating the absorbed dose delivered to liver metastases from colorectal cancer via ^90^Y-microspheres. QVH analyses could be used in combination with DVH to enhance the predictive value of ^99m^Tc-MAA SPECT/CT dosimetry and to assist personalized activity prescription.

**Supplementary Information:**

The online version contains supplementary material available at 10.1186/s40658-020-00345-4.

## Background

Radioembolization using ^90^Y-labelled microspheres injected in the intrahepatic tumour-feeding arteries is an effective loco-regional therapeutic procedure for non-resectable and chemoresistant liver metastases from colorectal cancer (mCRC) [1]. However, in a first-line setting, a large randomized multicentre trial showed no significant difference in terms of progression-free or overall survival for the combination of radioembolization with FOLFOX versus FOLFOX alone [2]. The latter trial used a simplified activity prescription model based on body surface area [3]. To address these unsatisfactory results, optimized activity prescription strategies considering patient-specific tumour and liver characteristics have been proposed [4–7].

The relationship between the mean absorbed dose delivered to the tumour and the outcome in mCRC patients has been demonstrated in several studies [6, 8, 9]. However, a high variability in tumour metabolic response evaluated by ^18^F-FDG PET/CT data was observed for the mean absorbed doses between 39 and 60 Gy [8]. This indicates that dose effects on tumour cells are not fully deterministic in this range, which is mostly due to the intra-tumour heterogeneity of ^90^Y-microspheres absorbed dose deposition, and that other parameters should be taken into account [8].

In this context, how well the ^99m^Tc-labelled macro-aggregates of albumin (^99m^Tc-MAA) predicts the absorbed dose delivered by radioembolization is essential. Even though multiple studies confirmed the good agreement of the predictive ^99m^Tc-MAA-SPECT/CT-based and the post-treatment dosimetry, based on parameters derived from dose-volume histograms (DVH) [10–13], some investigations showed mixed results. A good concordance between predictive and post-treatment dosimetry for the non-tumoural liver was reported in several studies, with more variable results for metastatic tumours [14, 15].

However, the use of DVH-derived parameters is limited by their lack of local information. In the published literature on the dose-painting paradigm from external beam radiation therapy (EBRT), the quality-volume histogram (QVH) has been proposed in some studies as a metric to compare planned and achieved dose for an inhomogeneous prescription inside the target volume [16, 17]. This method was introduced in 2006 by Vanderstraeten et al. and relies on the distribution of the voxel-based ratio between planned and prescribed dose within the tumour volume in head and neck cancer [18]. When the planned dose distribution perfectly matches the prescribed dose distribution, the ratio is equal to 1 for every voxel within the given volume. To the best of our knowledge, QVHs have not yet been applied and characterized in radioembolization dosimetry.

This work aimed to analyse the differences between ^99m^Tc-MAA-SPECT/CT and ^90^Y-microsphere PET/CT dosimetries at different levels, including the introduction of a voxel-to-voxel comparison using the QVH concept. QVH analysis was additionally used to demonstrate how it could be used to identify potential clinical parameters that are responsible for quantitative discrepancies between ^99m^Tc-MAA-SPECT/CT predictive and ^90^Y-microsphere-PET/CT post-treatment dosimetry.

## Materials and methods

### Patient selection and preparation

This single-institution, retrospective trial enrolled 29 patients with liver-only mCRC treated between January 2013 and September 2019 with resin ^90^Y-microspheres (SIR-Spheres, Sirtex medical Ltd., Sydney, Australia). The inclusion criteria were as follows: 18 years of age or older, histologically confirmed mCRC, unresectable, liver-only disease, chemorefractory, ECOG performance status < 2, adequate liver function without ascites and the same catheter position between simulation and treatment (assessed by an interventional radiologist). The exclusion criteria were as follows: different types of targeting (whole liver, lobar or segmental) at the simulation compare to treatment, prior radioembolization or external beam radiotherapy, chemotherapy within the last 4 weeks before radioembolization and second active cancer and extra-hepatic disease. The Jules Bordet Institute Ethics Committee approved this trial (CE2654). For this retrospective study, formal consent was not required.

Workup and treatment were performed following the current standard of practice in accordance with the manufacturer’s instructions [19–21]. Different types of targeting (segmental, lobar or whole liver) were used among patients. The activity of ^90^Y-microspheres to administer was determined using the partition model [5, 22]. The ^90^Y-microsphere activity was prepared in accordance with the prescription and was measured with the radionuclide calibrator (CRC-15R Capintec®, Florham Park, NJ, USA). Dose rates of the injection box were measured before and after the injection (all materials, including catheter, used to inject ^90^Y-microspheres were placed within the injection box) to evaluate the residual activity and compute the net administered activity.

### Image acquisition and reconstruction

Supplementary material 1 provides a complete description of the image acquisition and reconstruction process. Patients with clearly visible respiration motion artefacts in the hepatic region were excluded from the analysis.

### ^18^F-FDG-PET/CT lesion delineation

Recently, several studies demonstrated that derived biomarker and metabolic response were strongly correlated to clinical outcome in mCRC patients [6, 23–25]. Therefore, ^18^F-FDG-PET/CT is a suitable imaging technique for lesion identification and delineation. All ^18^F-FDG-PET/CT images were analysed using dedicated commercial software (PET VCAR v.4.6; Advantage Workstation; GE Healthcare®). Non-tumoural liver (NTL) background ^18^F-FDG uptake was determined by drawing a reference volume as a 3-cm diameter spherical volume of interest (VOI) located in the healthy liver parenchyma. Lesions were delineated using a fixed threshold corresponding to the PERCIST criteria for the identification of target lesion: 1.5 × SUV_mean_(NTL) + 2SD(NTL) and based on a method previously described [5, 8, 26]. ^18^F-FDG uptake bridging between two or more lesions was manually corrected. The maximal number of target lesions per patient was not restricted, and an experienced nuclear medicine physician blinded to the patients’ clinical and outcome data validated all delineations. Finally, the ^18^F-FDG-PET/CT was anatomically rigidly registered to the ^90^Y-PET/CT using Planet Onco (v3.0, Dosisoft®, Cachan, France) and lesion contours were mapped to the latter image set.

### ^99m^Tc-MAA-SPECT/CT and ^90^Y-PET/CT analyses

The Planet Onco clinical workstation was used for additional VOI delineation and predictive/post-treatment dose matrix generation.

The entire liver was manually delineated on the ^90^Y-PET/CT and the tumoural liver (TL, the Boolean union of all lesions VOIs) and NTL (Boolean subtraction of TL VOI from whole liver VOI) contours were defined.

Post-treatment voxel-based time-integrated activity matrix (TIA-matrix) for ^90^Y-microspheres was derived from ^90^Y-PET/CT datasets, using the ^90^Y-microsphere activity concentration directly quantified on the ^90^Y-PET/CT dataset.

Independently from the post-treatment TIA-matrix, the pre-treatment voxel-based TIA-matrix was derived from ^99m^Tc-MAA-SPECT/CT using the ^90^Y half-life and the net ^90^Y-microsphere administered activity determined at the end of the treatment (the difference between prepared and residual activities within the injection box).

Corresponding predictive and post-treatment ^90^Y-microsphere dose distribution matrices (D^Predictive^ and D^Post-treatment^) were finally and independently generated [5, 8].

All the dose distributions and corresponding CTs were exported and used as input in a processing workflow together with the previously segmented VOIs (liver, lesions, TL and NTL).

### Dose-matrices processing

**Fig. 1:**
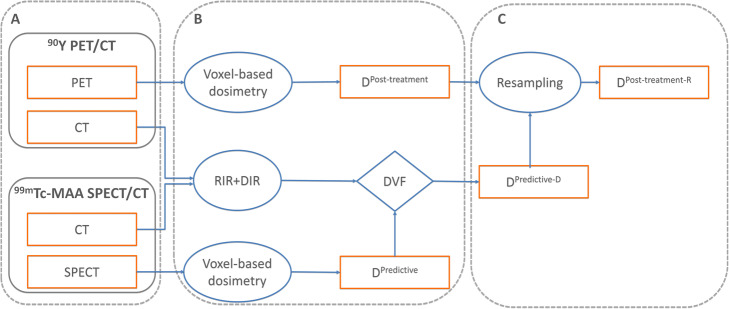
Schematic representation of the post-processing workflow. **a** ^90^Y-PET/CT and ^99m^Tc-MAA SPECT/CT images. **b** Dose matrices computed using the Planet Onco clinical workstation. Deformable image registration (DIR) and generation of the deformable vector field (DVF) using the validated HybridReg software. The deformation vector field (DVF) combines image information (i.e. intensities) with anatomical information provided by contoured image sets. The CT from the ^99m^Tc-MAA-SPECT/CT was non-rigidly registered to the CT from the ^90^Y-PET/CT and using the liver VOIs as deformation constrain. Image intensity-based deformations were therefore restricted to remain within the corresponding liver VOI representations, while outside intensities were discarded. To minimize deformation-induced modifications of D^Predicitve^ voxel values, the deformation knot grid and the MAA image resolution [cm/voxel] were kept around the same order (~ 0.5 cm/voxel). **c** Application of the DVF to the D^Predicitve^ (called D^Predicitve-D^). The D^Post-treatment^ was resampled (D^Post-treatment-R^) to the grid of D^Predicitve-D^ to accomplish voxel-to-voxel alignment (= identical grid position and spacing), using a trilinear interpolation method

Processing enabled normalizing all images to the same origin, rotation and scale. To achieve a voxel-to-voxel comparison, deformable image registration (DIR) was required. The determined deformation vector field (based on CT+VOI to CT+VOI co-registration) was applied to the D^Predicitve^ (called D^Predicitve-D^). Furthermore, the D^Post-treatment^ was resampled (D^Post-treatment-R^) to the grid of D^Predicitve-D^ to accomplish a voxel-to-voxel alignment (identical grid position and spacing). A complete description of dose-matrices processing can be found in Fig. 1. The processing workflow was performed using the MICE Toolkit 1.0.21-beta (Medical Interactive Creative Environment®), except for DIR that was determined using the clinically available HybridReg software (Raystation v7.0, RaySearch Laboratories®, Stockholm, Sweden), validated in the literature for different localizations (including the torso region) [27]. DIR was visually evaluated for smoothness and proper regularization using the DVF and grid. Additionally, the Jacobian determinant was calculated for each knob voxel. The Jacobian of the deformation field gives information about the image transformation consistency. For each patient, it was verified that the Jacobian determinant does not present negative values that would indicate folding of the deformation field [28].

### Dose-matrices analyses

All dose matrices were analysed using the MICE Toolkit software.

### Dose-volume histogram analysis

For all patients and all VOIs, DVHs were computed from D^Predicitve-D^ and D^Post-treatment-R^. For all DVHs, the following indices of interest were extracted: *D*_90_, *D*_70_, *D*_50_, *D*_mean_ and *D*_20_, where *D*_*X*_ represents the minimum dose received by at least *X*% of the given volume.

### Quality-volume histogram

#### Computation

QVHs were computed for each patient and each VOI, using an in-house Python code (available at https://github.com/hlevillain/Quality-volume-histograms) integrated into the MICE Toolkit as a plugin. The QVH represents the distribution of the voxel-based quality ratio, defined as:

1 $$ {Q}_i={\log}_{10}\left(\frac{D_i^{\mathrm{Post}-\mathrm{treatment}-\mathrm{R}}}{D_i^{\mathrm{Predictive}-\mathrm{D}}}\right) $$

where ${D}_i^{\mathrm{Post}-\mathrm{treatment}-\mathrm{R}}$ and ${D}_i^{\mathrm{Predictive}-\mathrm{D}}$ are respectively the post-treatment-resampled and predictive-deformed ^90^Y-microsphere doses in the *i*th voxel of a given VOI. QVH allows to visually and quantitatively assess the concordance between the predictive and post-treatment dosimetries. When the post-treatment dose perfectly matches the predictive dose in a given voxel, *Q*_*i*_ is equal to zero. Negative or positive *Q*_*i*_ correspond to voxels that received a higher or a lower absorbed dose at the predictive dosimetry compared to the post-treatment dosimetry. The logarithm function was required to have QVH symmetrical with respect to zero, facilitating its interpretation.

However, because of the mathematical definition of *Q*_*i*_, a discrepancy between two very low doses (< 10 Gy) or two high doses (> 200 Gy) at the predictive/post-treatment dosimetry leads to equivalent or even higher *Q*_*i*_ than two different doses comprised between 40 and 120 Gy. For instance, a voxel receiving 1 Gy at the predictive dosimetry and 3 Gy at the post-treatment dosimetry has a *Q*_*i*_ of *log*_10_3 ≈ 0.48, while a voxel receiving 50 Gy at the predictive dosimetry and 100 Gy at the post-treatment dosimetry has a *Q*_*i*_ of *log*_10_2 ≈ 0.30. The difference between 50 and 100 Gy is clinically much more important than the difference between 1 and 3 Gy and should thus give a higher *Q*_*i*_, not a lower one. Indeed a large *Q*_*i*_ for two very low doses (< 10 Gy) would not be considered clinically meaningful and could hinder accurate assessment of the agreement between predictive and post-treatment dosimetry. Previous studies demonstrated the existence of relationships between the lesion/non-tumoural liver *D*_mean_ and the response/toxicity in mCRC patients and showed that for *D*_mean_ < 10 Gy and > 200 Gy, the effects were not dose dependent (sigmoid functions) [6, 8, 9]. Therefore, weighting factors (*W*_*i*_) at the voxel scale have been introduced so that only volumes with clinically meaningful dose discrepancies are accounted for. In practice, voxels with a very low (< 10Gy) or very high (> 200 Gy) dose are given weighting factors *W*_*i*_(*P*) and *W*_*i*_(*T*) of zero to the predictive and post-treatment dose voxels, respectively. Dose differences considered as having the most clinical impact (between 40 and 120 Gy based on the *D*_mean_ – response/toxicity relationships) are given a *W*_*i*_(*P*) and a *W*_*i*_(*T*) of 1 [6, 8, 9]. Outside those ranges, *W*_*i*_(*P*) and a *W*_*i*_(*T*) are linearly interpolated. Supplementary material 2 illustrates the relationship between dose and *W*_*i*_. Since *W*_*i*_(*P*) and *W*_*i*_(*T*) are usually different, we set *W*_*i*_ = max(*W*_*i*_(*P*), *W*_*i*_(*T*)). However, in the extreme case where ${D}_i^{\mathrm{Post}-\mathrm{treatment}-\mathrm{R}}$ or ${D}_i^{\mathrm{Predictive}-\mathrm{D}}$was below 40 Gy while the other exceeded 120 Gy, such discrepancies must be highlighted, thus *W*_*i*_ = 1. *W*_*i*_ was applied on the voxel volume contribution into the QVH. Thus, a voxel with *W*_*i*_ = 1 contributes its entire volume to the QVH, and a voxel with *W*_*i*_ = 0 does not contribute at all to the QVH.

#### Quality factor

**Fig. 2:**
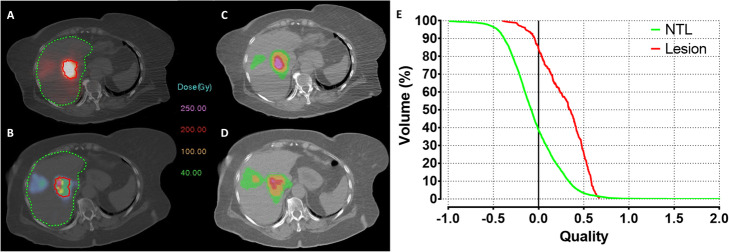
Axial images of a patient with liver mCRC and corresponding QVH. **a** Axial ^**90**^Y-PET/CT image on which target lesion and NTL volumes have been projected, showing a high ^**90**^Y-microsphere uptake within the lesion. **b** Corresponding axial ^99m^Tc-MAA SPECT/CT. **c**, **d** Post-treatment and predictive absorbed dose matrices. **e** Representation of QVH for both NTL and lesion with a QF of 0.24 and 0.32, respectively

Quality factors (QFs) were computed for all QVHs. The QF is a parameter used to summarize a QVH and thus the differences in terms of absorbed dose distributions between D^Predicitve-D^ and D^Post-treatment-R^. The QF is the absolute integral over the entire *Q*_*i*_ interval and is calculated by:

2 $$ \mathrm{QF}=\frac{\sum_i\left|{Q}_i\right|\times {W}_i}{\sum_i{W}_i} $$

where *i* ∈ {voxels}. The QF reflects the spread of the QVH from zero (ideal case). The QF of an ideal QVH is zero when the predictive dose distribution perfectly matches with the post-treatment. To facilitate the interpretation of results, a QF between 0 and 0.18 was considered as good, a QF between 0.18 and 0.3 was considered as acceptable and a QF > 0.3 was considered as poor. Figure 2 represents the ^90^Y-PET/CT and ^99m^Tc-MAA-SPECT/CT images with their corresponding dose matrices and QVHs, belonging respectively to acceptable and poor QF categorization.

### Statistics

Descriptive analyses were performed to summarize baseline patient characteristics and ^90^Y-microspheres predictive and post-treatment dosimetries. For each VOI category (NTL, TL and lesion), differences in extracted DVH indices between predictive and post-treatment dosimetries were investigated. All DVH index distributions were checked for normality, using the D’Agostino and Pearson normality test, and described with conventional statistics. Differences in extracted DVH indices between predictive and post-treatment dosimetries were compared using paired Student *t* test or Wilcoxon matched-pairs signed-rank test in case of non-normality. The first quartile, median and third quartile were computed to describe the QF of lesions, NTL and TL, respectively. Univariate associations between the explanatory variables: sex, age, the delay between predictive and post-treatment dosimetries, VOI volume, type of targeting (segmental, lobar or whole liver) and net administered activity, with the QF of the different VOIs were performed. Continuous variables were dichotomized around their median. Univariate differences in QFs for the explanatory variables and the different VOIs were assessed using unpaired Student *t* test or Mann-Whitney *U* test (in case of non-normality) for variables with two levels of categorization and with one-way ANOVA or Kruskal-Wallis test (in case of non-normality) for variables with three levels of categorization. Finally, to quantify the variation of dose only influenced processing the dose matrices, we calculated absorbed dose differences between original and processed DVHs (D^Predicitve^ vs. D^Predicitve-D^ and D^Post-treatment^ vs. D^Post-treatment-R^), NTL and TL overall patients. The statistical analyses were performed using the GraphPad software (v7.4; Prism®, La Jolla, CA, USA), and a two-tailed *p* value < 0.05 was considered statistically significant.

## Results

### Patients and treatment characteristics

Among the 29 patients included in this study, 3 patients were excluded from the analysis because at least one of their images was impaired by respiration artefacts. The remaining 26 patients had a median age of 73 years (range 45–83 years), and the median net administered activity was 1262 MBq (range 675–3314 MBq). The TL and NTL VOIs (*n* = 26) were obtained for all analysed patients, and 130 lesions were delineated on baseline ^18^F-FDG-PET/CT with a median number of 4 lesions per patient (range 1–9). Baseline characteristics are presented in Table 1.

**Table 1:** Baseline characteristics of patients

Characteristics	Value, *n* (%) or median (range)
**Sex**	
Male	12 (46%)
Female	14 (54%)
**Age (years)**	73 (45-83)
**Prior liver surgery**	6 (23%)
**Prior bevacizumab**	15 (58%)
**Lesion volume (ml)**	5.8 (1.8–17.57)
**Tumoural liver volume (ml)**	73 (7.8–201.5)
**Non-tumoural liver volume (ml)**	1354 (913–4267)
**Type of targeting**	
Whole liver, common hepatic artery	8 (30%)
Whole liver, left and right hepatic arteries	6 (23%)
Uni-lobar	12 (47%)
**Delay between predictive and post-treatment dosimetry (days)**	9 (6–33)
**Net administered activity (MBq)**	1262 (675–3314)

### DVH analyses

**Fig. 3:**
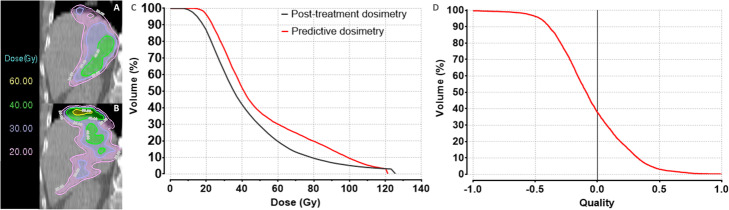
Example of discordance between DVHs and QVH analyses. **a** Coronal D^Post-treatment-R^ image, showing doses ranging from 20 to 40 Gy in the NTL. **b** Coronal D^Predicitve-D^ image, showing doses ranging from 20 to 60 Gy in the NTL. Areas covered by the different isodoses are similar, but their patterns are different. **c** DVHs obtained for patient’s NTL at predictive and post-treatment dosimetry, showing the similarity between the two dosimetries. **d** Corresponding QVH showing a poor agreement (QF = 0.34) between the two dosimetries

Table 2 summarizes the values of extracted DVH indices for both D^Post-treatment-R^ and D^Predicitve-D^ for the different VOIs (lesions, TL and NTL). An example of discordance between DVH and QVH analyses is presented in Fig. 3.

**Table 2:** DVH indices of D^Post-treatment-R^ and D^Predicitve-D^ for the different VOIs

DVH indices	D^Post-treatment-R^, value, median (interquartile range (IQR))	D^Predicitve-D^, value, median (IQR)	*p*
**Lesion**			
*D*_90_ (Gy)	47 (31–64)	45 (25–69)	0.88
*D*_70_ (Gy)	57 (40–77)	59 (36–94)	0.35
*D*_50_ (Gy)	63 (46–87)	73 (42–111)	0.16
*D*_mean_ (Gy)	61 (44–84)	65 (45–101)	0.16
*D*_20_ (Gy)	73 (56–102)	94 (55–139)	**0.0004**
**TL**			
*D*_90_ (Gy)	38 (21–56)	25 (16–56)	0.19
*D*_70_ (Gy)	56 (35–77)	47 (26–96)	0.26
*D*_50_ (Gy)	65 (48–103)	60 (42–122)	0.77
*D*_mean_ (Gy)	69 (47–96)	71 (45–113)	0.37
*D*_20_ (Gy)	100 (64–128)	108 (61–148)	0.25
**NTL**			
*D*_90_ (Gy)	10 (2–18)	5 (1–14)	**< 0.0001**
*D*_70_ (Gy)	20 (5–30)	16 (4–25)	**0.005**
*D*_50_ (Gy)	29 (16–39)	28 (11–39)	0.09
*D*_mean_ (Gy)	32 (23–41)	35 (22–43)	0.14
*D*_20_ (Gy)	52 (39–61)	58 (43–66)	0.06

### QVH analysis

**Fig. 4:**
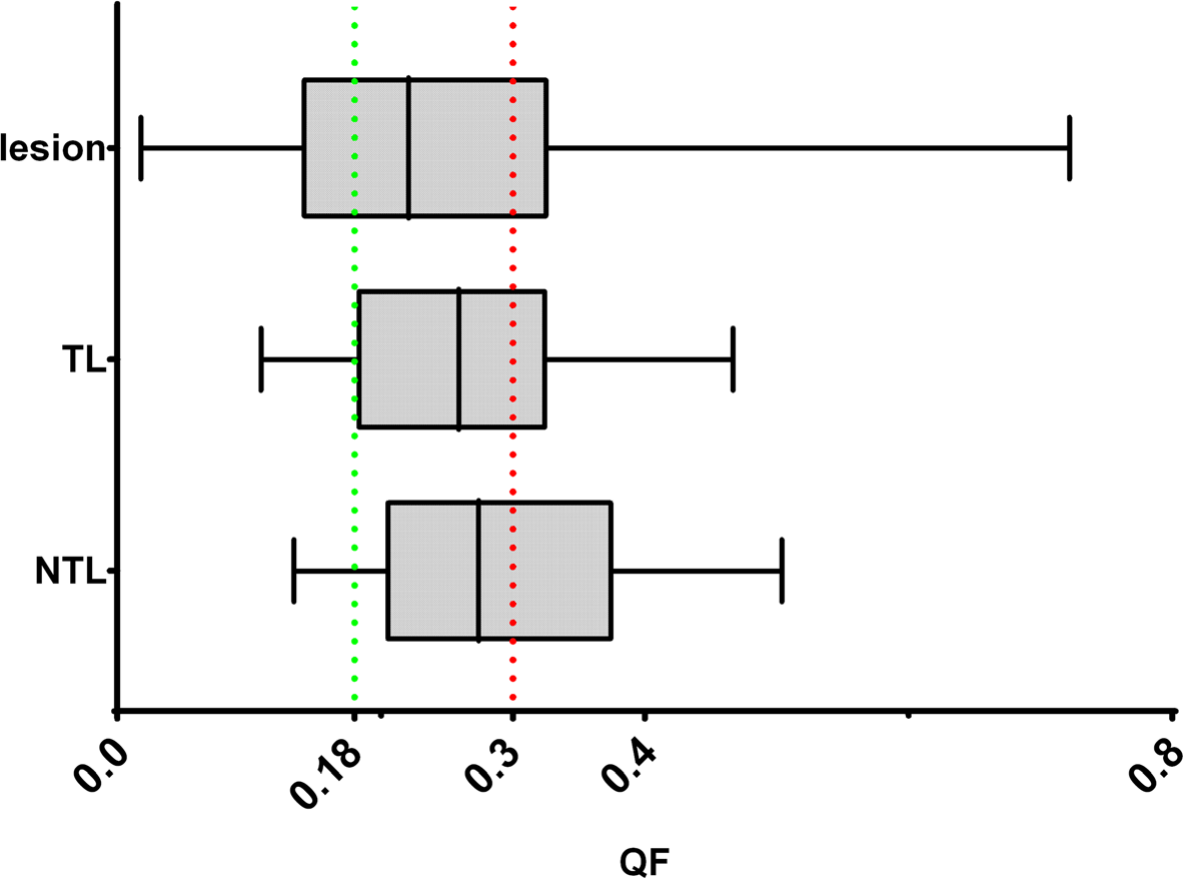
QF distribution for the different VOIs and the two defined cut-offs 0.18 and 0.3 which allow classification of QF values between good, acceptable and poor concordance

Figure 4 illustrates the QF distributions for the different VOIs. The median values and IQR were 0.22 (0.14–0.33), 0.26 (0.18–0.33) and 0.28 (0.21–0.38) for lesions, TL and NTL, respectively.

Table 3 presents the concordance rates between D^Post-treatment-R^ and D^Predicitve-D^ for all VOIs, based on their QF classification. Interestingly, 48% of patients showed poor agreement between D^Post-treatment-R^ and D^Predicitve-D^ at the NTL level.

**Table 3:** Concordance rates between D^Post-treatment-R^ and D^Predicitve-D^ for all VOIs, based on their QF classification

	Good (%)	Acceptable (%)	Poor (%)
Lesions (*n* = 130)	40	29	31
TL (*n* = 26)	23	42	35
NTL (*n* = 26)	12	40	48

**Fig. 5:**
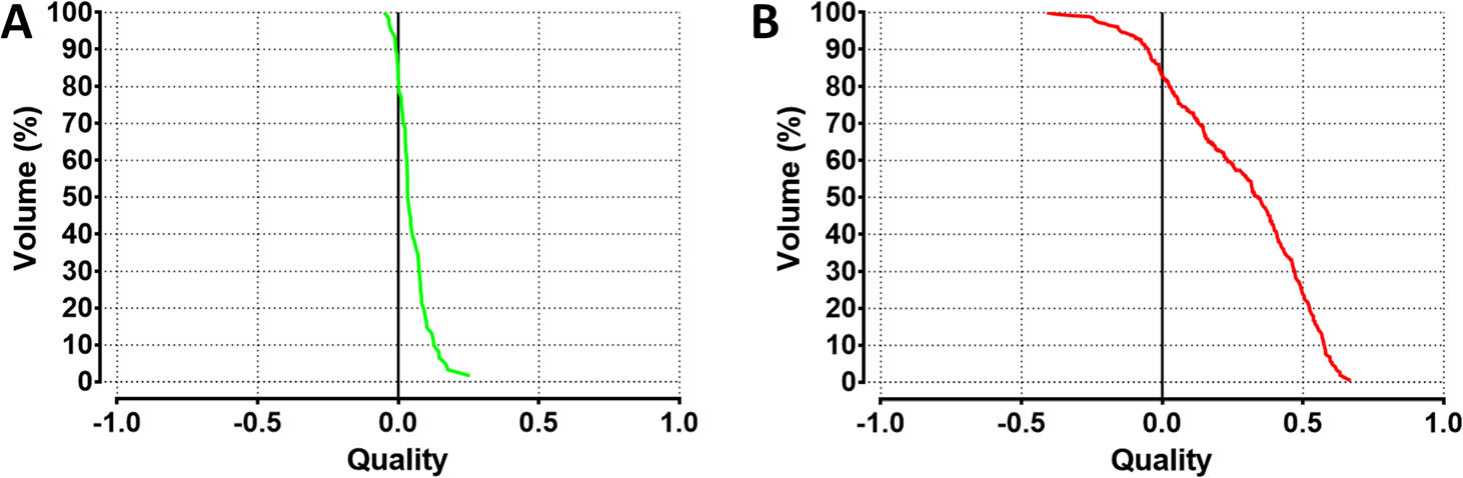
QVHs, belonging respectively to good and poor QF categorization. **a** QVH with a QF of 0.05 belonging to the good QF category. **b** QVH with a QF of 0.32 belonging to the poor QF category

Figure 5 illustrates the two QVHs, belonging respectively to good and poor QF categorization.

Table 4 summarizes the univariate associations of the dichotomized explanatory variables with QF values for different VOIs. The delay between predictive and post-treatment dosimetries was a significant predictor of D^Post-treatment-R^ and D^Predicitve-D^ differences across all VOIs with the cut-off at 9 days. Furthermore, the type of targeting (whole liver single injection, whole liver injection left and right separately and uni-lobar) showed significant differences for lesions (smaller lesions’ QF in whole liver injection and larger ones in uni-lobar injection).

**Table 4:** Univariate analysis of predictive factors of poor agreement between D^Post-treatment-R^ and D^Predicitve-D^ for lesion, TL and NTL

Variables	Dichotomisation	QF Lesion				QF TL				QF NTL			
		median (IQR)			*p*	median (IQR)			*p*	median (IQR)			*p*
Sex	Male vs. Female	0.23 (0.14-0.34)	vs	0.22 (0.13-0.33)	0.50	0.29 (0.20-0.37)	vs	0.25 (0.17-0.29)	0.22	0.27 (0.19-0.36)	vs	0.27 (0.22-0.40)	0.79
Age (y)	> 73 vs ≤73	0.21 (0.14-0.33)	vs	0.23 (0.14-0.34)	0.54	0.25 (0.16-0.33)	vs	0.26(0.20-0.34)	0.61	0.24 (0.19-0.43)	vs	0.28 (0.24-0.32)	0.47
Previous Liver surgery	Yes vs No	0.26 (0.15-0.34)	vs	0.22 (0.14-0.32)	0.74	0.28 (0.18-0.39)	vs	0.26 (0.17-0.30)	0.53	0.24 (0.20-0.33)	vs	0.28 (0.19-0.39)	0.70
Previous Bevacizumab	Yes vs No	0.19 (0.13-0.34)	vs	0.26 (0.17-0.33)	0.07	0.24 (0.17-0.33)	vs	0.27 (0.19-0.33)	0.98	0.27 (0.18-0.33)	vs	0.28 (0.23-0.43)	0.19
Delay predictive / post-treatment dosimetry (d)	> 9 vs ≤9	0.26 (0.17-0.38)	vs	0.19 (0.12-0.30)	**0.003**	0.28 (0.22-0.37)	vs	0.22 (0.15-0.27)	**0.03**	0.33 (0.28-0.41)	vs	0.22 (0.18-0.28)	**0.004**
Net administered activity (MBq)	> 1262 vs ≤1262	0.20 (0.13-0.34)	vs	0.24 (0.16-0.33)	0.49	0.25 (0.20-0.35)	vs	0.26 (0.17-0.33)	0.64	0.26 (0.19-0.35)	vs	0.28 (0.21-0.41)	0.50
Lesion Volume (ml)	> 5.8 vs ≤5.8	0.23 (0.15-0.32)	vs	0.21 (0.13-0.34)	0.51								
TL Volume (ml)	> 73.34 vs ≤73.34					0.28 (0.16-0.37)	vs	0.26 (0.20-0.30)	0.40				
NTL Volume (ml)	> 1354 vs ≤1354									0.29 (0.21-0.41)	vs	0.27 (0.20-0.31)	0.37
Type of targeting	Whole liver single injection	0.17 (0.12-0.28)			**0.02**	0.20 (0.15-0.28)			0.65	0.23 (0.17-0.33)			0.42
	Whole Liver injection left and right lobes separately	0.24 (0.14-0.35)				0.27 (0.17-0.37)				0.31 (0.19-0.41)			
	Uni-lobar	0.26 (0.18-0.33)				0.27 (0.22-0.33)				0.28 (0.24-0.38)			

### Processing influence on dose matrices

**Fig. 6:**
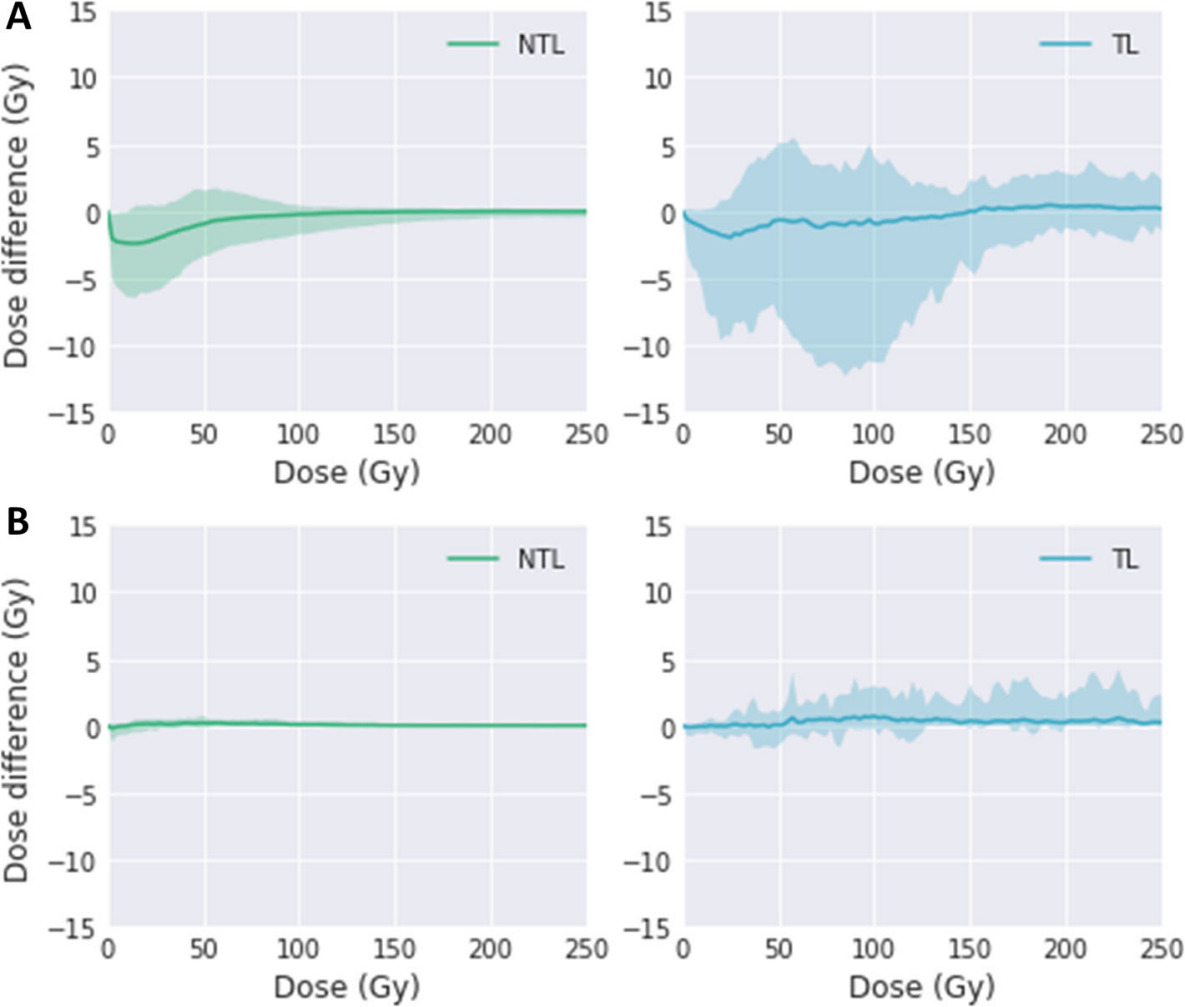
The mean value (solid line) and associated 95% CI (shaded area) of dose differences before and after processing for NTL and TL. Dose differences were calculated as a function of dose bins (1 Gy/bin) ranging from 0 to 250 Gy. **a** Dose differences between original and deformed D^Predicitve^. **b** Dose differences between original and resampled D^Post-treatment^

Figure 6 presents the mean dose difference due to the deformation (D^Predicitve^ to D^Predicitve-D^) and resampling (D^Post-treatment^ to D^Post-treatment-R^) together with 95% CI for NTL and TL for all patients. The largest differences for the deformations were up to 10 Gy for TL, while it remained less than 6 Gy for NTL. Resampling showed smaller differences with a maximum of 4 Gy for TL across the whole dose range and negligible (< 1 Gy) differences for NTL.

## Discussion

This work aimed to analyse the differences between ^99m^Tc-MAA-SPECT/CT and ^90^Y-microsphere PET/CT dosimetries at different levels, including the introduction of a voxel-to-voxel comparison using the QVH concept. DVHs are extremely useful to compare dose plans; however, other evaluation criteria can be considered to complement and improve the situations. A major drawback of the DVH method is the lack of spatial information of dose distributions, i.e. DVHs do not show where within a specific volume a dose is received [29]. Therefore, comparing post-treatment and predictive DVHs of the same VOI, to assess the agreement between D^Predicitve-D^ and D^Post-treatment-R^ heterogeneity, may be insufficient. Two similar DVHs could correspond to different spatial dose distributions. However, QVHs are based on a voxel-by-voxel comparison and deal with dose differences directly. Thus, even though a QVH does not give either complete spatial information, it compares voxels at the same location and is therefore suited to assess D^Predicitve-D^ and D^Post-treatment-R^ heterogeneity differences (see Fig. 3). A future perspective would be to display the value of the *Q*_*i*_ of each voxel to obtain a *Q*_*i*_ map, which would show the location of the difference. Recently, Ferreira et al. investigated the value of ^99m^Tc-MAA-SPECT/CT-based predictive dosimetry, using the gamma-index (γ-index) analytical method [30]. This γ-index defines a combined evaluation of geometrical (distance to agreement) and dosimetric (dose difference) accuracy. In their paper, the acceptance criteria generally used in EBRT were also adapted in the case of radioembolization, passing rates > 90% were achieved for 15 mm/15% tolerance criteria. Importantly, several studies in EBRT demonstrated that gamma analysis was not correlated to the clinical impact of a dose discrepancy [31, 32]. Furthermore, the γ-index is taking into account at the same time spatial and dose discrepancies; thus, the outcome of the evaluation does not provide sufficient precision for very small lesions. In our case, we overcome the spatial mismatch by performing an oriented DIR based on the CT and liver delineation information. This way, our main investigation could solely focus on the dose differences. The proposed QVH method is not intended to replace the DVH and γ-index analyses, but to bring additional information to assess the compliance between the radioembolization predictive and post-treatment dose distribution.

Our results confirmed that the implementation of QVH from EBRT into radioembolization is feasible and gives complementary information to the DVH-based analysis for the comparison of predictive and post-treatment ^90^Y-microsphere radioembolization dosimetry. The QF provides a rapid and easy interpretation of the agreement between predictive and post-treatment dosimetries.

In contrast to dose-painting in EBRT for target volume, absorbed dose ranges encountered in radioembolization are much wider, with voxel doses ranging from 0 to 250 Gy or higher, including target and normal tissue VOIs. Therefore, the QVH model from EBRT must be adapted to the context of radioembolization. Notably, the QVH used in EBRT is calculated from the direct dose ratio while we introduced the log of the ratio. Since dose differences can be much higher in radioembolization than in EBRT, the ratio alone would have produced very asymmetrical QVHs. In particular, doses infinitely higher in the predictive dose matrix compare to post-treatment dose matrix would produce a QVH tending to zero and a QF tending to one. On the contrary, doses infinitely higher on the post-treatment dose matrix compared to the predictive dose matrix would have led to both the QVH and QF tending to infinity. Two such extreme scenarios should produce the same outcome: a QF demonstrating a poor concordance. The introduction of the log of the ratio makes the QVH symmetrical with respect to zero, and both scenarios then yield a very large QF.

Also, dose differences between very low doses (< 10 Gy) or high dose (> 200 Gy) would have led to high *Q*_*i*_ (e.g. a voxel receiving 1 Gy at the predictive dosimetry and 3 Gy at the post-treatment dosimetry has a *Q*_*i*_ of log_10_3 ≈ 0.48) and would therefore highlight a large discrepancy between predictive and post-treatment dosimetry, while it would not be considered as such in clinical practice. Besides, this could hinder accurate assessment of the agreement between predictive and post-treatment dosimetry, as a discrepancy between two very low doses (< 10 Gy) or two high doses (> 200 Gy) at the predictive/post-treatment dosimetry leads to equivalent or even higher *Q*_*i*_ than two different doses comprised between 40 and 120 Gy. In our previously published report, post-treatment absorbed dose cut-offs of 60 Gy and 40 Gy for predicting respectively metabolic response and non-response were also defined [8]. Similarly, absorbed dose > 50 Gy and > 40–60 Gy also provided better metabolic response in two studies [6, 9]. In these trials, lesions that received more than 100–120 Gy had a higher probability of complete metabolic response. Therefore, weighting factors were defined for each voxel and according to clinical data in mCRC patients, to limit the influence of low and extremely high doses on the QVH shape [6, 8, 9]. This parameter should be adapted in case glass spheres are used for the radioembolization (as their specific activity differs from that of resin spheres) and/or with other types of liver cancer [33]. The *W*_*i*_ was applied to the voxel volume contribution into the QVH. Other options would have been to apply directly the *W*_*i*_ to the *Q*_*i*_ or not to apply *W*_*i*_ factors at all. Further research using patient clinical outcome is needed to decide on the best approach.

To facilitate interpretation of QVHs and QFs, we introduced cut-offs to classify QVHs into good (QF < 0.18), acceptable (0.18 ≤ QF < 0.3) and poor (QF ≥ 0.3). Admittedly, these cut-offs were arbitrarily defined as a first-estimate analysis. Cut-offs of 0.18 and 0.3 correspond to cases where one of the dose maps is systematically 33% and 50% lower than the other, respectively. Further research is needed to define cut-offs linked with clinical outcomes (prediction of treatment failure and disease-free survival).

Therefore, because of the assumption made on the weighting factors and the arbitrary categorization of QFs, the clinical conclusion derived from the QVH analysis is limited.

DVH results showed good agreement between D^Predicitve-D^ and D^Post-treatment-R^ for individual lesions, whole tumoural liver (TL) and non-tumoural liver (NTL). Notably, the DVH analysis showed no significant difference in terms of *D*_mean_, confirming results from previous studies [10, 11]. Hence, previously defined ^90^Y-PET/CT-based *D*_mean_ cut-offs may be used at the predictive dosimetry for determining the activity of ^90^Y-microspheres to administer [6, 8, 9]. Statistically significant dose differences between predictive and post-treatment dosimetries were found in NTL for *D*_90_ and *D*_70_ (10 vs. 5 Gy, *p* < 0.0001 and 20 vs. 16 Gy, *p* = 0.005, respectively), but can be considered clinically not significant.

For lesions and TL, DVH and QVH results are mostly concordant. In terms of dose distribution correspondence assessed with QVH, 69% of lesions had a QF < 0.3 (40% < 0.18) and 65% of TL had a QF < 0.3 (23% < 0.18). These results suggest that dose heterogeneity in lesion and TL could be reasonably predicted by the ^99m^Tc-MAA predictive dosimetry. Interestingly, several studies suggested that the lesions/TL minimal dose (*D*_min_) would be an interesting parameter to take into consideration for activity prescription, to ensure that the entire volume receives at least *D*_min_ [8, 34]. Our results, and principally DVH analysis, support that it would be possible.

On the other hand, DVH and QVH analyses of the NTL showed mixed results. In the QVH analysis, only 12%/40% of NTL comparisons resulted in good/acceptable agreement, while 48% showed poor agreement between D^Post-treatment^ and D^Predicitve^. Therefore, QVH findings brought additional information to the DVH analysis and highlighted that NTL dose heterogeneity on the post-treatment dosimetry might differ from the one predicted by D^Predicitve^. This should be taken into consideration in ongoing and future clinical trials aiming to define new NTL dose cut-offs and/or to combine D^Predicitve^ heterogeneity with liver function, e.g. assessed with 3D hepatobiliary-scintigraphy, to predict treatment toxicity [35, 36]. These differences in dose heterogeneity can be partly explained by the difference between the number of administered ^90^Y-microspheres and ^99m^Tc-MAA. Recently, Walrand et al. showed that the physical embolization redirected a part of the resin microspheres to other parts of the arterial tree because of the high number of microspheres (40–80 million). As the capillaries of the tumours are progressively embolized (due to the high number of resin microspheres) during the administration, the redirection will increase and transport more resin ^90^Y-microspheres into the NTL. On the contrary, the physical embolization and redirection with glass spheres were negligible because of their lower number (1.2 million), which is comparable with the MAA particle number (2–4.5 million) [33, 37]. Therefore, differences in dose distribution in the NTL could be explained by the much higher number of resin ^90^Y-microspheres, within the NTL compared to ^99m^Tc-MAA. Other possible sources of difference in dose distribution between predictive and post-treatment dosimetries are that some of the MAA particles are smaller than the microspheres (10–70 μm and 20–60 μm, respectively), which can lead to different distribution patterns and shunt to extrahepatic organ and consequently to different dose deposition [37]. Also, because of the degradation of MAA in the liver, the dissociated ^99m^Tc-pertechnetate can hinder an accurate evaluation of the dose distribution [38]. Importantly, all patients were orally administrated with 1 mL sodium perchlorate (Irenat, Alliance Pharmaceutical®, Chippenham, UK) to avoid dissociated ^99m^Tc-pertechnetate uptake to non-targeted organs (gastric region, thyroid gland). These effects will also impact the dose distribution within the lesions and especially larger ones. However, because of the vascular properties and the smaller volume of the tumour in mCRC patients, the impact of these effects on the differences between predictive/post-treatment dosimetry will be lower than for the NTL.

QVHs could be also used to identify predictive factors for the differences between D^Predicitve^ and D^Post-treatment^. In our study, a delay between predictive and post-treatment dosimetry > 9 days was associated with a significantly higher QF across all VOIs. Importantly, the 9 days cut-off used in this study was the median value and was not derived through a method to optimize the predictive power, such as a receiver operating characteristic curve, that could be part of further work. Radinsky et al. demonstrated that mCRC expresses high levels of vascular endothelial growth factor promoting angiogenesis and tumour growth, contributing to their relatively poor prognosis [25, 39]. QVHs could be used to define a maximal delay between predictive and post-treatment dosimetry to limit anatomical/vascularization modification caused by disease progression and thereby to maximize the conformity of dose distributions. The catheter tip position variation between predictive and post-treatment dosimetry has also been reported as a critical variable predictive of dose differences [11]. In this report, only patients with the same catheter position between predictive and post-treatment dosimetry (assessed by an interventional radiologist) were included.

The combination of DVH and QVH analyses allows a more extensive assessment of radioembolization. This quality assurance process could have two different impacts:

Firstly, even if the evaluation per patient is de facto performed after the treatment, the results of this study support that optimizing radioembolization activity prescription using ^99m^Tc-MAA dose heterogeneity is feasible in patients with liver mCRC. Notably, *D*_min_ could be used instead of *D*_mean_, to ensure that the entire volume will be sufficiently treated. Additionally, QVH analysis could be used to identify factors impacting the agreement between predictive and post-treatment dosimetry such as the delay between the two dosimetries. Nevertheless, this would need to be investigated in future trials. Therefore, the results of this quality assurance process could benefit to future patients by assessing the entire predictive value of pre-treatment dosimetry which could enable to use it at its full potential for personalizing the activity of ^90^Y-microspheres to administer.

Secondly, it can be used to determine for a specific patient if the post-treatment dosimetry was performed in concordance with the predictive dosimetry and therefore with the therapeutic intent (pre-operative setting, bridging to surgery or palliative setting). Performing this quality assurance process compels the clinicians to compare predictive and post-treatment dosimetry and, in the case of large discrepancies, it can alert clinicians and could streamline the decision to retreat. Indeed, for example, in case post-SIRT dosimetry shows that lesions were underdosed completely or partially in comparison with what was planned based on the predictive dosimetry, clinicians could then identify the possible reason for the discrepancy (e.g. different catheter positioning). A new treatment could be then proposed and adapted with this information. Or in case of a NTL overdose, clinicians can decide to monitor liver toxicity more closely.

Ultimately, both the quality assurance process, based on combined DVH and QVH analyses, and an enhanced personalized radioembolization could contribute to a better patient outcome.

The end-to-end processing time for QVH calculation and evaluation is around 20 min. Several software packages were used to implement the QVH method (Fig. 1) as at the time of this investigation, no single solution was able to cover the entire process. Importantly, dose-matrices computation and DIR were obtained using clinically available software. Performance and quality of the DIR algorithm are essential, as QVH computation required voxel-to-voxel association. Therefore, the DIR was performed using the HybridReg solution, which was validated for several regions (including the torso region) [27]. To have the same voxel size between dose matrices D^Post-treatment^ (pixels of 2.73 × 2.73 mm with a slice thickness of 3.27 mm) was resampled to the grid of D^Predicitve^ (pixels of 4.79 × 4.79 mm with a slice thickness of 4.79 mm), which consequently reduced miss-registration errors. Also, we chose the largest available (5 mm) isotropic knob size for the deformation, to avoid any overfitting and limit the freedom of the DIR. Finally, the overall induced differences between original/processed dose matrices were within clinically acceptable limits (Fig. 4). Notably, dose differences before vs. after DIR of the predictive dose matrix were up to 10 Gy for TL. To increase the speed and improve the reproducibility of the method, it would be required to implement the entire process into a single software and to make an extensive analysis of the DIR performance. This will be advocated before using QVH in clinical routine and/or in multicentre trials.

Several limitations of this study should be noted. The study is subject to bias, due to its retrospective character. Also, the small sample size limits the generalizability of our results to other datasets. The number of lesions per patient was not restricted. The impact of acquisition and reconstruction parameter on QVH results was not tested but should be an important point to investigate in a future trial. Even though several actions were undertaken to maximize the performance of the DIR, its influence on QVH results should be further evaluated. Weighting factors should be adapted in case glass spheres are used and/or with other types of liver cancer. This study did not include any clinical outcome to conclude on the real influence of a good/bad matching between predictive and post-treatment dosimetry on patients. Notably, cut-offs for classifying QFs into good, acceptable and poor categories were defined arbitrarily which influences our results. Further studies should intend to better define them. Thus, the clinical conclusions derived from the QVH analysis are limited. Finally, our method and results must be validated in prospective multicentre studies.

## Conclusion

The QVH approach was introduced and adapted for radioembolization dosimetry, and the feasibility of this approach was tested retrospectively. In unresectable and chemorefractory liver-only mCRC patients, the combination of DVH and QVH analyses showed promising results on the predictive value of ^99m^Tc-MAA SPECT/CT predictive dosimetry, for personalizing radioembolization activity prescription. This study also highlighted the potential additional value of the QVH analysis. The combination of DVH and QVH analyses could be a powerful tool for the quality assurance process in mono/multi-centre trials as well as in clinical routine especially when its relationship with patient outcomes is established.

## Supplementary Information

**Additional file 1: Supplementary material 1.** Image acquisition and reconstruction parameters.

**Additional file 2: Supplementary material 2.** Relationship between dose and weighting factors.

## Data Availability

The datasets used and/or analysed during the current study are available from the corresponding author on reasonable request.

## Abbreviation

CI: Confidence interval
CT: Computed tomography
EBRT: External beam radiation therapy
ECOG: Eastern Cooperative Oncology Group
DIR: Deformable image registration
DVH: Dose-volume histograms
D^Predicitve^: Predictive ^90^Y-microsphere dose matrix
D^Predicitve-D^: D^Predicitve^-Deformed
D^Post-treatment^: Post-treatment ^90^Y-microsphere dose matrix
D^Post-treatment-R^: D^Post-treatment^-Resampled
IQR: Interquartile range
mCRC: Metastases from colorectal cancer
NTL: Non-tumoural liver
PET: Positron emission tomography
QF: Quality factor
QVH: Quality-volume histogram
^99m^Tc-MAA: ^99m^Tc-labelled macro-aggregates of albumin
TIA-matrix: Time-integrated activity matrix
TL: Whole tumoural liver
VOI: Volume of interest
*W*_*i*_: Weighting factors for voxel i
