# Correction to: Combined quality and dose-volume histograms for assessing the predictive value of ^99m^Tc-MAA SPECT/CT simulation for personalizing radioembolization treatment in liver metastatic colorectal cancer

**DOI:** 10.1186/s40658-021-00356-9

**Published:** 2021-01-27

**Authors:** Hugo Levillain, Manuela Burghelea, Ivan Duran Derijckere, Thomas Guiot, Akos Gulyban, Bruno Vanderlinden, Michael Vouche, Patrick Flamen, Nick Reynaert

**Affiliations:** 1 Medical Physics Department, Jules Bordet Institute, Université Libre de Bruxelles, 1 Rue Héger-Bordet, B-1000 Brussels, Belgium; 2 Nuclear Medicine Department, Jules Bordet Institute, Université Libre de Bruxelles, 1 Rue Héger-Bordet, 1000 Brussels, Belgium; 3 Department of Radiology, Jules Bordet Institute, Université Libre de Bruxelles, 1 Rue Héger-Bordet, 1000 Brussels, Belgium

**Correction to: EJNMMI Phys 7, 75 (2020)**

**https://doi.org/10.1186/s40658-020-00345-4**

Following publication of the original article [1], it was noted that, due to a typesetting error, Table 4 was missing several columns.

**Table 4:** Univariate analysis of predictive factors of poor agreement between D^Post-treatment-R^ and D^Predicitve-D^ for Lesion, TL and NTL

Variables	Dichotomisation	QF Lesion		QF TL		QF NTL	
		median (IQR)	*p*	median (IQR)	*p*	median (IQR)	*p*
Sex	Male vs. Female	0.23 (0.14-0.34) vs 0.22 (0.13-0.33)	0.50	0.29 (0.20-0.37) vs 0.25 (0.17-0.29)	0.22	0.27 (0.19-0.36) vs 0.27 (0.22-0.40)	0.79
Age (y)	> 73 vs ≤73	0.21 (0.14-0.33) vs 0.23 (0.14-0.34)	0.54	0.25 (0.16-0.33) vs 0.26 (0.20-0.34)	0.61	0.24 (0.19-0.43) vs 0.28 (0.24-0.32)	0.47
Previous Liver surgery	Yes vs No	0.26 (0.15-0.34) vs 0.22 (0.14-0.32)	0.74	0.28 (0.18-0.39) vs 0.26 (0.17-0.30)	0.53	0.24 (0.20-0.33) vs 0.28 (0.19-0.39)	0.70
Previous Bevacizumab	Yes vs No	0.19 (0.13-0.34) vs 0.26 (0.17-0.33)	0.07	0.24 (0.17-0.33) vs 0.27 (0.19-0.33)	0.98	0.27 (0.18-0.33) vs 0.28 (0.23-0.43)	0.19
Delay predictive / post-treatment dosimetry (d)	> 9 vs ≤9	0.26 (0.17-0.38) vs 0.19 (0.12-0.30)	**0.003**	0.28 (0.22-0.37) vs 0.22 (0.15-0.27)	**0.03**	0.33 (0.28-0.41) vs 0.22 (0.18-0.28)	**0.004**
Net administered activity (MBq)	> 1262 vs ≤1262	0.20 (0.13-0.34) vs 0.24 (0.16-0.33)	0.49	0.25 (0.20-0.35) vs 0.26 (0.17-0.33)	0.64	0.26 (0.19-0.35) vs 0.28 (0.21-0.41)	0.50
Lesion Volume (ml)	> 5.8 vs ≤5.8	0.23 (0.15-0.32) vs 0.21 (0.13-0.34)	0.51				
TL Volume (ml)	> 73.34 vs ≤73.34			0.28 (0.16-0.37) vs 0.26 (0.20-0.30)	0.40		
NTL Volume (ml)	> 1354 vs ≤1354					0.29 (0.21-0.41) vs 0.27 (0.20-0.31)	0.37
Type of targeting	Whole liver single injection	0.17 (0.12-0.28)	**0.02**	0.20 (0.15-0.28)	0.65	0.23 (0.17-0.33)	0.42
	Whole Liver injection left and right lobes separately	0.24 (0.14-0.35)		0.27 (0.17-0.37)		0.31 (0.19-0.41)	
	Uni-lobar	0.26 (0.18-0.33)		0.27 (0.22-0.33)		0.28 (0.24-0.38)	

The corrected Table 4 has been reproduced in this Correction article and the original article has been updated.
