# Supplementary material for: Combined quality and dose-volume histograms for assessing the predictive value of 99mTc-MAA SPECT/CT simulation for personalizing radioembolization treatment in liver metastatic colorectal cancer
Source: EJNMMI Phys. 2020 Dec 14;7:75. doi: 10.1186/s40658-020-00345-4 (PMC7736450; doi:10.1186/s40658-020-00345-4)
Supplement: Supplementary file 1 — Additional file 1: Supplementary material 1. Image acquisition and reconstruction parameters. [file 40658_2020_345_MOESM1_ESM.pdf]

Article title

Combined Quality and Dose Volume Histograms for assessing the predictive value of ^{99m}Tc-MAA SPECT/CT simulation for personalizing radioembolization treatment in liver metastatic colorectal cancer

Journal name

European Journal of Nuclear Medicine and Molecular Imaging Physics

Authors and affiliations

Hugo Levillain[1,2], Manuela Burghelea[1], Ivan Duran Derijckere[2], Thomas Guiot[1], Akos Gulyban[1], Bruno Vanderlinden[1], Michael Vouche[3], Patrick Flamen[2], Nick Reynaert[1]

1 Medical Physics Department, Jules Bordet Institute, Université Libre de Bruxelles, 1 rue Héger-Bordet, 1000 Brussels, Belgium.

2 Nuclear Medicine Department, Jules Bordet Institute, Université Libre de Bruxelles, 1 rue Héger-Bordet, 1000 Brussels, Belgium.

3 Department of Radiology, Jules Bordet Institute, Université Libre de Bruxelles, 1 Rue Héger-Bordet 1000 Brussels, Belgium

Corresponding author

Hugo Levillain

hugo.levillain@bordet.be

Caption

Supplementary material 1: Image acquisition and reconstruction parameters

All baseline ^{18}F-FDG-PET/CT images were acquired with EARL-approved settings to guarantee image quality standardisation. As other centres referred some patients, several PET/CT systems were involved. Most of the ^{18}F-FDG-PET/CT images were acquired on a General Electric (GE) Discovery 690 time-of-flight PET system (matrix size was 192 × 192 pixels of 2.73 × 2.73 mm with a slice thickness of 3.27 mm; reconstruction: 3D OSEM algorithm with 2 iterations and 18 subsets, post-filtering : 6.4 mm FWHM Gaussian function). For two patients, ^{18}F-FDG-PET/CT images Siemens Biograph64-mCT (matrix size was 192 × 192 pixels of 3.65 × 3.65 mm with a slice thickness of 3.0 mm; reconstruction: 3D OSEM algorithm with 4 iterations and 8 subsets). And for one patients, ^{18}F-FDG-PET/CT images were acquired using a Siemens Biograph128-mCT (matrix size was 200 × 200 pixels of 4.07 × 4.07 mm with a slice thickness of 3.26 mm; reconstruction: 3D OSEM algorithm with 2 iterations and 21 subsets). Patients were required to have fasted for at least 6 h and to have blood glucose levels < 150 mg/dL before FDG injection. Images were acquired 60 min (range: 60–70 min) after injection of 4.4 MBq/kg (range: 3.6–4.8 MBq/kg) (at injection time). Attenuation and scatter corrections were applied on all images.

^{99m}Tc-MAA-SPECT/CT images were acquired 50 min (range: 26-80 min) after injection of 186 MBq (range: 75-301 MBq) of ^{99m}Tc-MAA. All patients were injected with MAASOL (GE Healthcare®): 2,0x10^{6} ± 15% MAA particles per vial, except for 1 patient who was injected with LyoMAA (Curium®): 4.5x10^{6} ± 15% MAA particles per vial. All ^{99m}Tc-MAA-SPECT/CT images were acquired using a Siemens Symbia T system with LEHR collimators and auto-contour mode, with an acquisition time of 20 min (32 projections, 30 s per projection) and a matrix of 128 × 128 pixels of 4.79 × 4.79 mm with a slice thickness of 4.79 mm. Attenuation and scatter corrections were applied on all images. Images were reconstructed with a 3D ordered subset expectation maximisation (OSEM) algorithm with 8 iterations and 8 subsets, and were post-filtered with a 4.8 mm full width at half maximum (FWHM) Gaussian function.

^{90}Y-PET/CT imaging was performed on average 21 h (range: 20–23 h) after ^{90}Y-microsphere administration using a GE Discovery 690 TOF PET system, in 3D mode. Acquisition time was 1 h (30 min per bed position, two bed positions with an overlap of 13 slices) and matrix size was 192 × 192 pixels of 2.73 × 2.73 mm with a slice thickness of 3.27 mm. Attenuation, scatter and resolution recovery (VPFX) corrections were applied. Images were reconstructed with a 3D OSEM algorithm with 18 iterations and 3 subsets, and were post-filtered with a 13.7 mm FWHM Gaussian function.
