# Supplementary material for: Combined quality and dose-volume histograms for assessing the predictive value of 99mTc-MAA SPECT/CT simulation for personalizing radioembolization treatment in liver metastatic colorectal cancer
Source: EJNMMI Phys. 2020 Dec 14;7:75. doi: 10.1186/s40658-020-00345-4 (PMC7736450; doi:10.1186/s40658-020-00345-4)
Supplement: Supplementary file 2 — Additional file 2: Supplementary material 2. Relationship between dose and weighting factors. [file 40658_2020_345_MOESM2_ESM.pdf]

Article title

Combined Quality and Dose Volume Histograms for assessing the predictive value of ^{99m}Tc-MAA SPECT/CT simulation for personalizing radioembolization treatment in liver metastatic colorectal cancer

Journal name

European Journal of Nuclear Medicine and Molecular Imaging Physics

Authors and affiliations

Hugo Levillain[1,2], Manuela Burghelea[1], Ivan Duran Derijckere[2], Thomas Guiot[1], Akos Gulyban[1], Bruno Vanderlinden[1], Michael Vouche[3], Patrick Flamen[2], Nick Reynaert[1]

1 Medical Physics Department, Jules Bordet Institute, Université Libre de Bruxelles, 1 rue Héger-Bordet, 1000 Brussels, Belgium.

2 Nuclear Medicine Department, Jules Bordet Institute, Université Libre de Bruxelles, 1 rue Héger-Bordet, 1000 Brussels, Belgium.

3 Department of Radiology, Jules Bordet Institute, Université Libre de Bruxelles, 1 Rue Héger-Bordet 1000 Brussels, Belgium

Corresponding author

Hugo Levillain

hugo.levillain@bordet.be

Caption

Supplementary material 2: Relationship between dose and weighting factors

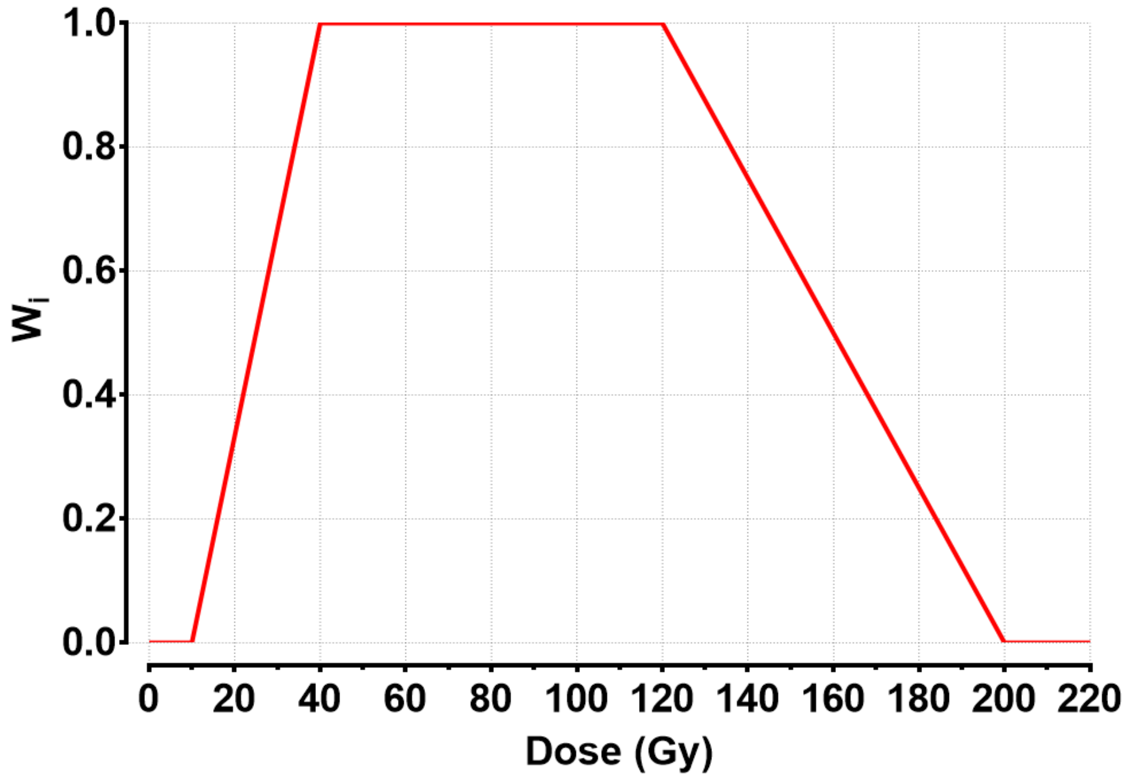

1.0
0.8
0.6
0.4
0.2
0.0
W_i
0
20
40
60
80
100
120
140
160
180
200
220
Dose (Gy)
